# Supplementary material for: Assessing the relative contributions of mosaic and regulatory developmental modes from single-cell trajectories
Source: PLoS Comput Biol. 2025 Dec 15;21(12):e1012352. doi: 10.1371/journal.pcbi.1012352 (PMC12721551; doi:10.1371/journal.pcbi.1012352)
Supplement: S1 Fig — The values are mildly positive. The raw correlations are hardly interpretable because of the mixed effects of lineage, spatial positions and context. This justifies to build a pipeline to disentangle the various sources of correlation. (PDF) [file pcbi.1012352.s001.pdf]

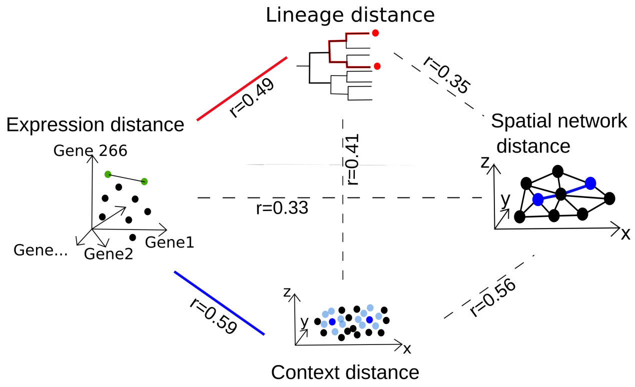


**S1 Fig: Pearson correlation r between all pairs of measured distances** The values are midly positive. The raw correlations are hardly interpretable because of the entanglement of the effects of lineage, spatial positions and context. This justifies to build a pipeline to disentangle the effects.
