# Supplementary material for: Assessing the relative contributions of mosaic and regulatory developmental modes from single-cell trajectories
Source: PLoS Comput Biol. 2025 Dec 15;21(12):e1012352. doi: 10.1371/journal.pcbi.1012352 (PMC12721551; doi:10.1371/journal.pcbi.1012352)
Supplement: S2 Fig — Two cells which are close in terms of spatial positions might share a certain proportion of their respective neighbors. When this is the case, the context distance between them will be low just because most of their neighbors are common. Here we plot A) the proportion of shared neighbors between two cells with respect to the physical distance between them. The physical distance is defined as the length of the shortest path between them in the Delaunay graph of all cells nuclei positions. The proportion of shared neighbors is calculated as the Jaccard index between the respective sets of neighbors of the two cells. B)The average and standard deviation of Jaccard index for all pairs at a given time point. (PDF) [file pcbi.1012352.s002.pdf]

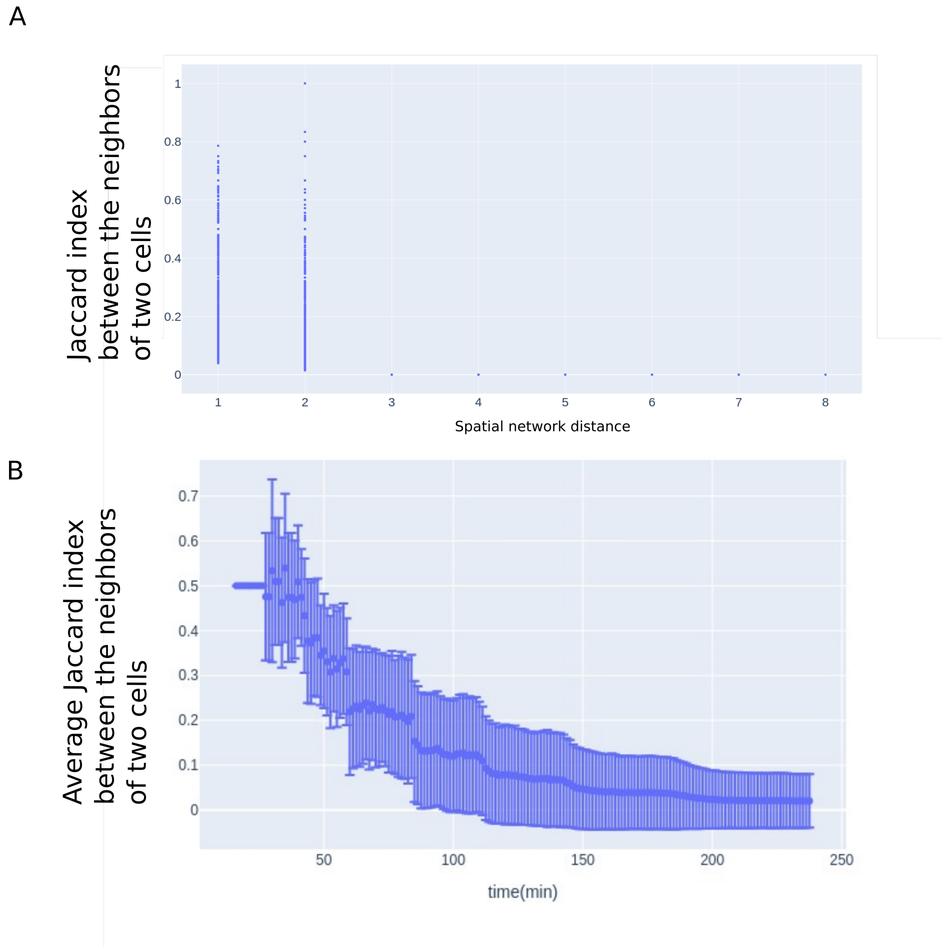

S2 Fig: Proportion of shared neighbors between two cells
Two cells which are close in terms of spatial positions might share a certain proportion of their respective neighbors. When this is the case, the context distance between them will be low just because most of their neighbors are common. Here we plot A) the proportion of shared neighbors between two cells with respect to the physical distance between them. The physical distance is defined as the length of the shortest path between them in the Delaunay graph of all cells nuclei positions. The proportion of shared neighbors is calculated as the Jaccard index between the respective sets of neighbors of the two cells. B)The average and standard deviation of Jaccard index for all pairs at a given time point.
