# Supplementary material for: Assessing the relative contributions of mosaic and regulatory developmental modes from single-cell trajectories
Source: PLoS Comput Biol. 2025 Dec 15;21(12):e1012352. doi: 10.1371/journal.pcbi.1012352 (PMC12721551; doi:10.1371/journal.pcbi.1012352)
Supplement: S3 Fig — Each row shows the same analysis as for Figure 3, performed by retaining only the pairs of cells that belong to a given tissue, in the sense that they belong to lines that lead to terminal cells labeled as belonging to this tissue. (PDF) [file pcbi.1012352.s003.pdf]

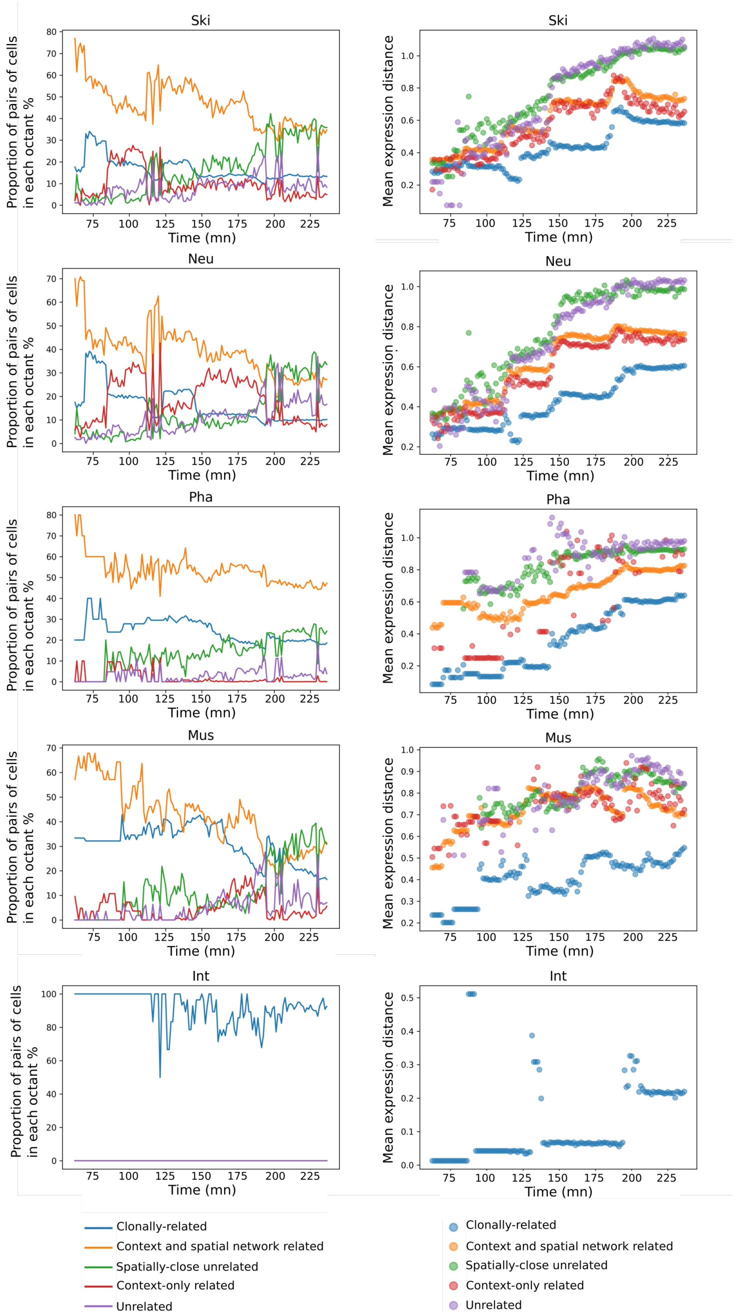

S3 Fig: Octant analysis of each tissue separately in *C. elegans* data Each row shows the same analysis as for Figure 3, performed by retaining only the pairs of cells that belong to a given tissue, in the sense that they belong to mother to daughter lines that lead to terminal cells labeled as belonging to this tissue.
