# Supplementary material for: Assessing the relative contributions of mosaic and regulatory developmental modes from single-cell trajectories
Source: PLoS Comput Biol. 2025 Dec 15;21(12):e1012352. doi: 10.1371/journal.pcbi.1012352 (PMC12721551; doi:10.1371/journal.pcbi.1012352)
Supplement: S4 Fig — Same analysis as in Fig 3 performed on the lineage-defined model and the random null model. (PDF) [file pcbi.1012352.s004.pdf]

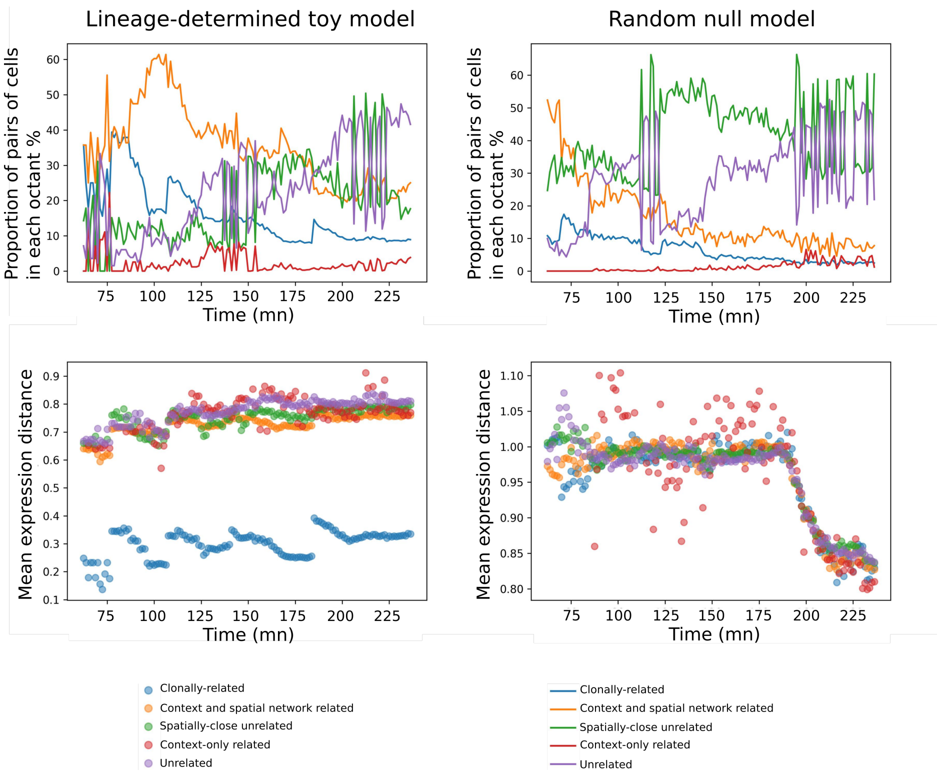

S4 Fig: Octant analysis for the artificial models

Same analysis as in Figure 3 performed on the lineage-defined model and the random null model.
