# Supplementary material for: Assessing the relative contributions of mosaic and regulatory developmental modes from single-cell trajectories
Source: PLoS Comput Biol. 2025 Dec 15;21(12):e1012352. doi: 10.1371/journal.pcbi.1012352 (PMC12721551; doi:10.1371/journal.pcbi.1012352)
Supplement: S5 Fig — Distributions of each of the four types of distances at different time: lineage distances, context distances, expression distances and physical distances. The distributions are asymmetric and their shapes vary with time. (PDF) [file pcbi.1012352.s005.pdf]

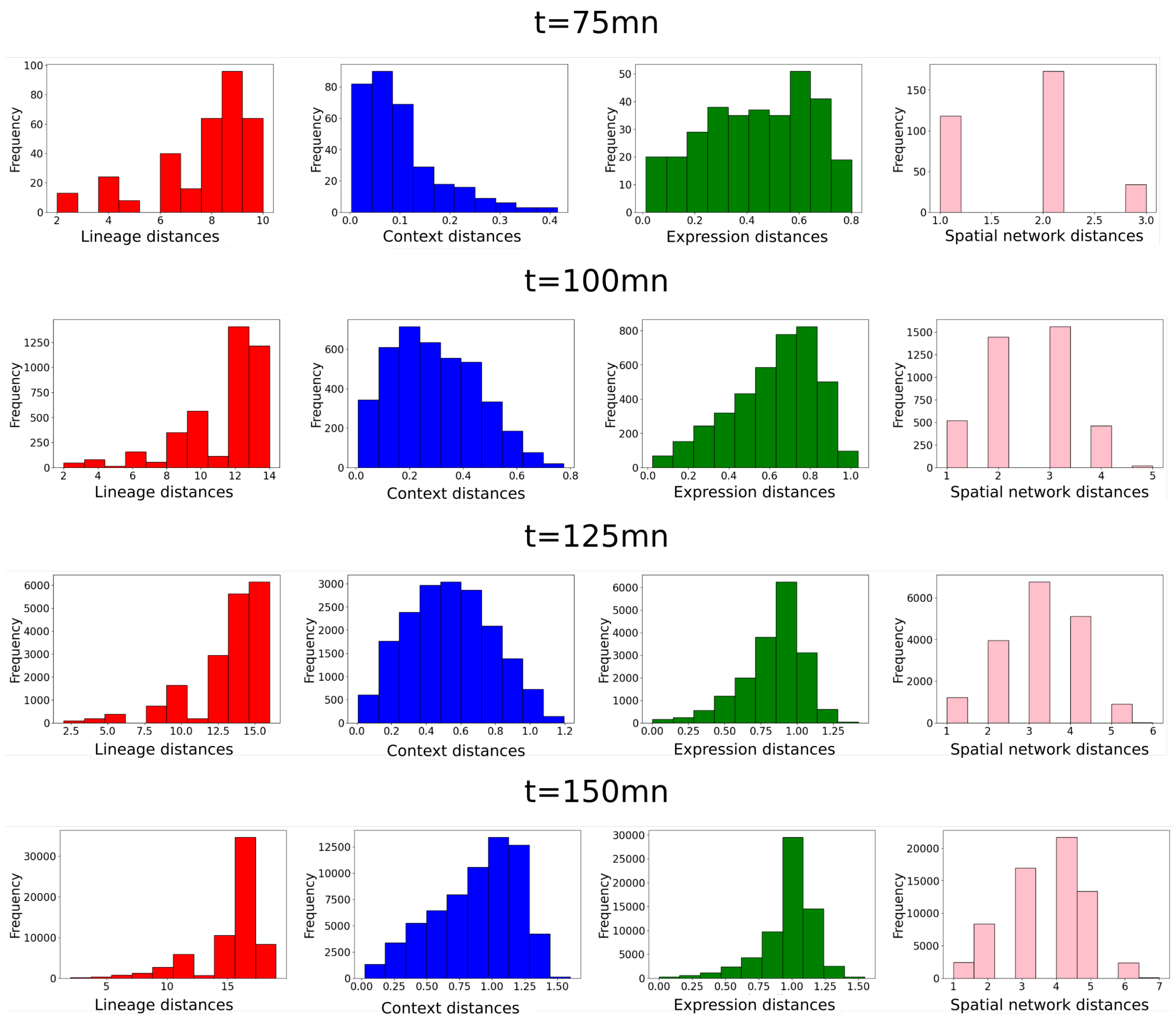

S5 Fig: Distributions of each of the four types of distances

Distributions of each of the four types of distances at different time: lineage distances, context distances, expression distances and spatial network distances. The distributions are asymmetric and their shapes vary with time.
