# Supplementary material for: Assessing the relative contributions of mosaic and regulatory developmental modes from single-cell trajectories
Source: PLoS Comput Biol. 2025 Dec 15;21(12):e1012352. doi: 10.1371/journal.pcbi.1012352 (PMC12721551; doi:10.1371/journal.pcbi.1012352)
Supplement: S6 Fig — The spatial distribution of cells belonging to each labelled tissue at t = 237 mn. At this time step, which is the last one in which the spatial positions are tracked, the cells are not yet all fully differentiated so that some cells may appear more than one tissue if their daughters have different tissue types. Cell types visualization with UMAP. (PDF) [file pcbi.1012352.s006.pdf]

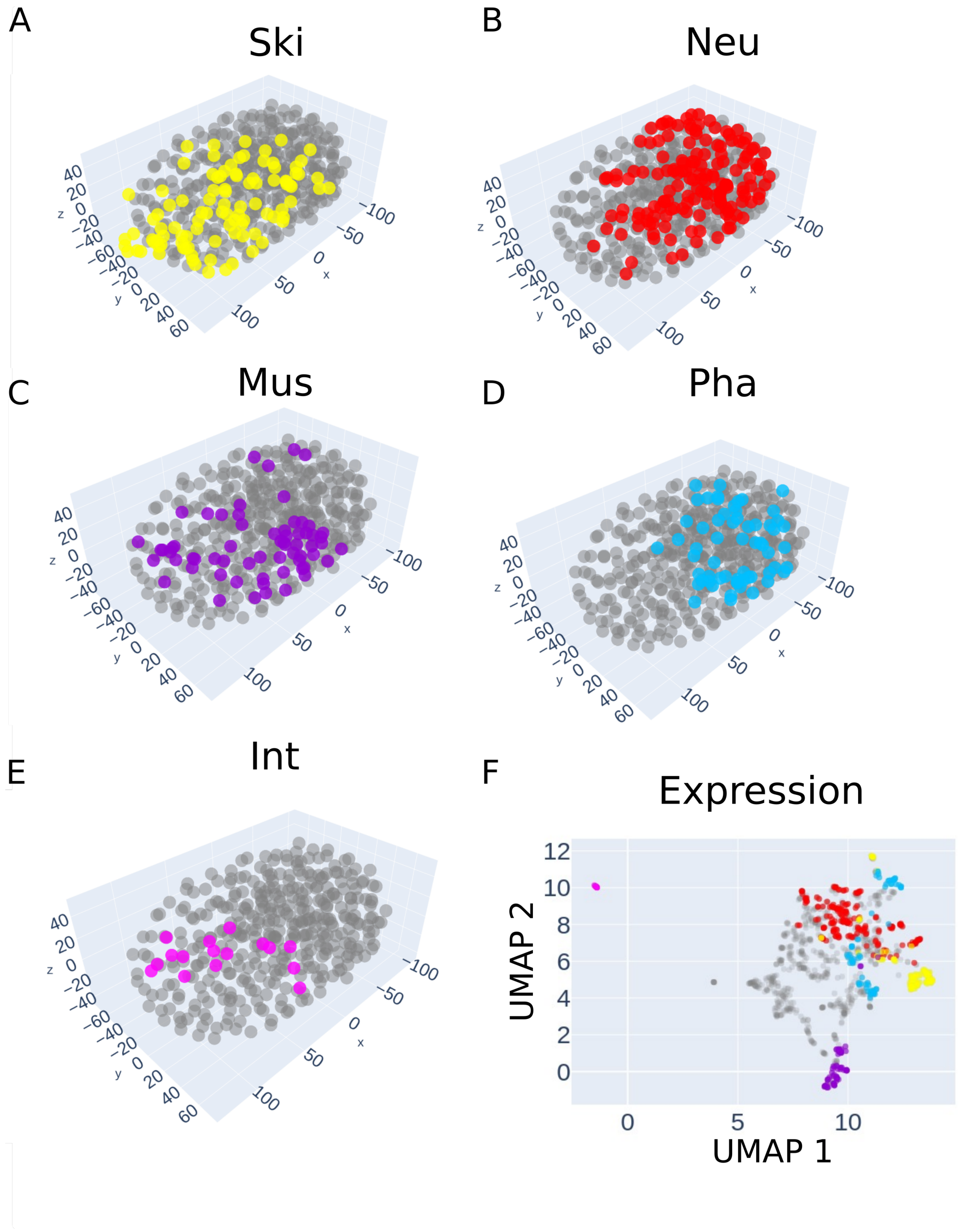

S6 Fig Tissues labels visualization: The spatial distribution of cells belonging to each labelled tissue at t = 237 mn. At this time step, which is the last one in which the spatial positions are tracked, the cells are not yet all fully differentiated so that some cells may appear more than one tissue if their daughters have different tissue types. Cell types visualization with UMAP
