# Supplementary material for: Assessing the relative contributions of mosaic and regulatory developmental modes from single-cell trajectories
Source: PLoS Comput Biol. 2025 Dec 15;21(12):e1012352. doi: 10.1371/journal.pcbi.1012352 (PMC12721551; doi:10.1371/journal.pcbi.1012352)
Supplement: Attachment — Submitted filename: Answer to reviewers.pdf [file pcbi.1012352.s007.pdf]

Dear Editors,

Please find below the response to the reviewers' comments. We have made major revisions to the introduction, description of methods, and results. We've also expanded our analyses, in particular we've analyzed the "octants" results in the random null model, which provides additional support for our conclusions.

Sincerely,

Paul Villoutreix

Reviewer #1: In this article, the authors propose a new computational method to evaluate the contribution of mosaic and regulative modes to the development of an organism and apply it to an extensive dataset of C. elegans development. The authors first propose a measure for the contribution of the mosaic mode at a particular developmental time based on the correlation between cell-cell lineage distance and cell-cell expression distance, while the regulative mode contribution is measured as the correlation between cell-cell context distance and cell-cell expression distance. The authors leverage two extensive datasets of C. elegans development jointly containing, for each cell, information about their lineage, spatial positions and gene expression in time and use these measures to evaluate the contribution of mosaic and regulative modes during the development of different C. elegans tissues. The authors find that while the development of some tissues, such as the intestine, develop mainly through a mosaic mode, other tissues, such as the skin, have a higher mosaic contribution, particularly at the end of their development. The authors also study the effects of context, lineage, and spatial position on gene expression distance as well as the correlation between the changes in the modes and the proliferation and spatial rearrangements.

I believe there is a convincing motivation for the authors' study, and the two rich datasets used in the study form a great context to test and leverage their proposed method. I think their computational method is mostly sound, and their claims seem supported by their analyses.

We thank reviewer 1 for their positive assessment of our manuscript and we respond to the specific comments below.

A possible caveat of their method, which the authors mention in the Discussion section, is that the authors evaluate the contribution of each mode by a measure of correlation between the gene expression distance at time t, and the lineage or context distance at time t. However, one could imagine that the effect of the context, lineage, or spatial position in the gene expression, and particularly cell fate, could take longer than 75 seconds (I am not sure if this is true in C. elegans in particular, but it is in other organisms). I still think, however, that their approach is relevant and a first step toward a more general method.

We had to make a trade-off between the different timescales, fast and small for molecular processes and slower and larger for tissue-level processes. By combining two data sets, we combine two timescales. On the one hand, the spatial information is obtained from time-lapse movies with a temporal resolution of 75 seconds, while on the other hand, the protein expression atlas provides information for each cell in the lineage, averaged over the entire cell cycle, thus with a coarser temporal resolution of several tens of minutes. The effect of context, lineage, or spatial position is therefore examined across these two timescales. An analysis of molecular processes at the 75-second resolution would require the generation of new data with adapted live fluorescence microscopy imaging, at the expense of the number of protein reporters measured simultaneously.

Having said this, I have several concerns about the current state of the manuscript, which I detail below:

Major comments

1.- The authors utilize two datasets that record spatial position and gene expression data every 75 seconds for 6 hours, which I believe would result in around 288 time points. However, in the Datasets section of the paper, lines 110 and 112, figures, and the rest of the manuscript, the authors mention that the time points range from t=0 to t=190. Could the authors clarify how they got to that number? I believe this is important, as all the conclusions depend on this dataset.

We thank reviewer 1 for noticing this, an error was made in reporting the number of hours. We do have 190 time points with 75s, but this results in 4 hours instead of 6 hours. We corrected the sentence. To avoid any doubt on the timing, we converted all the time steps into minutes in the manuscript.

2.- I believe the text and organization of the manuscript need some revision:
- I would suggest the authors revise the writing and figure labels as I have seen quite a few typos (two “the” on line 17, “it’s” instead of “its” on line 106, “build” instead of “built” on line 149, “replacing inverting” on line 177,…).

These typos have been fixed. Thank you for the careful reading.

- In the second subsection of the Results section, the introduction of the 8 quadrants seemed a bit abrupt, while the third subsection introduces this quantification in great detail. Also, in the second subsection, the authors mention “unrelated pairs of cells” (line 348), which is not defined until the third subsection. Have the authors considered changing the order of these subsections?

This is a good suggestion and we have now reversed the order of these two subsections for ease of reading.

- In lines 381-382 the authors write “This analysis by configuration can also be done for each

tissue separately, which gives additional insights as shown in supplementary." I haven't been able to find any supplementary information attached to the submission, could the authors clarify what this supplementary is?

Indeed, this supplementary figure was missing in the initial submission. We added it. It is now supplementary figure 3.

- In lines 415-417 the authors briefly mention the behavior of muscle cells as opposed to such of skin cells in how proliferation and spatial rearrangements change the modes of specification. Could the authors expand on how the differences in divisions and rearrangements between muscle and skin cells translate into different modes and whether their model can give an explanation for this difference? Do the authors suggest that exclusive regulative mode needs for high proliferation and rearrangement rate?

We mean that proliferation and rearrangement are two mechanisms that give alternative explanations to a high $W_{regulative}$ rate. Indeed, high $W_{regulative}$ can be interpreted as the fact that expression distances are highly and robustly correlated with context distances. The first explanation for this is that newly divided cells have adopted the same identities as cells which are located in the same context, which is what we seeked to measure originally. The second explanation is that cells that are already similar in expression migrate to join similar contexts. This second explanation for a high $W_{regulative}$ does not really illustrate regulative mode for cell fate determination but rather cell sorting. In the case of Skin, at the end of tracked development, when the $W_{regulative}$ is high, the two mechanisms are at play successively: within the phase of high $W_{regulative}$, there is one proliferation phase, followed by a rearrangement phase. In the particular case of Skin, based on the timing, we guess that this captures the end of gastrulation and the beginning of cells movements in the epidermal cells, starting with the closure of ventral cleft which starts at 230 minutes:

http://www.wormbook.org/chapters/www_epidermalmorphogenesis/epidermalmorphogenesis.html.

We added a sentence in the results section to add this information.

We show the Muscle case as an example in which the separation of both phases (proliferative and rearrangement) is not clear because the divisions are more asynchronous. We added a sentence at the end of the paragraph to precise why we oppose the muscle and skin.

- In Figure 2, the continuous and dashed lines are said to be the intrinsic variation in the shuffled toy model, do the authors mean the null or the lineage toy model?

We meant the null model. We changed the "shuffled" for "random null" in the corrected manuscript.

- The title of Figure 3D appears cut.

Indeed. We changed the figure.

- Legend of Figure 3C, could the authors clarify what “the tracks in 3D space” mean? Are they the spatial coordinates of the two cells in time?

In Figure 5C, yes, it is the spatial coordinates of the two selected cells, and their successive ascendent cells (mother, grand-mother, etc). We added this precision in the legend.

3.- When the authors study the effect of the context, independently of lineage or spatial relationships, they state that Figure 3 shows that the red quadrant, where the cells have similar context but are distant in space and lineage, “comprises between 10 and 50% of cells along time points between t=50 and t=190” (lines 337-339). However, in Figure 3C, if I understand it correctly, the red line is always below 30%. Could the authors clarify this inconsistency?

Indeed, this is an error in the text. It is between 10% and 30%. We corrected it in the manuscript.

Minor comments

1.- The authors measure rearrangements as the Jaccard distance between the list of neighbors at time t and at time t-1. How do the authors deal with cell divisions where the neighbors at time t and t-1 are different cells? I might not have understood how they measure rearrangements, so I would really appreciate if they could expand on their method.

When a cell in the neighbors list at time t-1 divides into two daughter cells at time t, the two daughter cells are both considered different than the mother cell. We have expanded on the measure of rearrangement in the revised manuscript in the Material and Methods section, and we have corrected a typo on the expression of the Jaccard index, where the | | symbol for number of elements in the set was initially misplaced.

2.- When the authors compare the contributions of each mode in each tissue, they explain that they “consider as being part of a tissue all the cells that belong to tracks leading to the leaves that are labeled as this tissue”, lines 301-303. However, all the cells in the organism come from the first single cell, and also, there might be progenitors that give rise to two tissues eventually. How do the authors deal with this issue? Do the authors start the quantifications shown in Figure 2 from the first cell quantified or from the first progenitor that exclusively gives rise to that tissue?

We consider as being part of a tissue all the cells that belong to tracks leading to the leaves that are labeled as this tissue. With this criterion, most cells belong to multiple tissues, with the extreme case of the first single cell P0 which belongs to all tissues

3.- When the authors study the effect of the context, independently of lineage or spatial

relationships, they classify the data across quadrants using the mid-distance between the minimal and maximal value of each type of distance (lines 331-332). I was wondering if the authors have considered any other distance more stable to the presence of outliers, such as the median of each type of distance.

Thank you for the suggestion. Yes, we have considered other thresholds such as median or mean. However, given that the shapes of the distributions of distances vary a lot from a time point to another, and from a type of distance to another, we decided to choose the threshold which depends least on the shape of the distributions, which is the mid distance between the extreme values, which equals to make a binning of 8 bins of the data points. We added as a supplementary figure the distances distributions at four different time points to show how they differ. We added a paragraph in the Octants definition section of the Methods section to explain this point.

Reviewer #2: In this work, the authors studied the two modes of specifying cell types in development, i.e., mosaic and regulative modes. Specifically, they aim to quantify the respective contributions of the two models from single-cell data for C. elegans. By calculating some measured defined by the authors and correlation analysis, they demonstrate the co-existence of mosaic and regulative modes in development of C. elegans. Some specific conclusions include that in the skin during early development, the mosaic mode dominates while at later stages, regulative mode dominates.

Overall, although I appreciate the efforts to using single cell data to explore the cell fate determination problem, I didn't see much new insight from this paper from the perspective of cell fate decision mechanism. A major problem is that the authors show many different observations from statistics of data, but the underlying mechanism, especially the one related with cell fate decision, a core problem in systems biology, is mostly not explored.

We thank Reviewer 2 for their careful reading and assessment of our manuscript, we updated the manuscript according to their recommendation. The initial question that we asked, “is development mosaic or regulative” is very general and encompasses a multitude of molecular mechanisms in each species, cell types, periods of development. We propose here to address this question in a mechanisms-agnostic manner in order to draw conclusions that are as general as possible. Going further, we could compare the statistics of mosaic versus regulative modes in various species, starting for example with close relatives of *C. elegans* such as *C. briggsae* (e.g. in the paper: “Lineage-resolved analysis of embryonic gene expression evolution in C. elegans and C. briggsae Large” *et al., bioRxiv* 2024). This could reveal highly regulated mosaic periods of development versus more robust regulative period. For this reason of generalizability, we chose to focus on pairwise differences between cells and not on the protein levels themselves (the features).

Please find below our answer to each of the specific points.

1, The two modes proposed, mosaic mode and regulative mode, seem related to the two

mechanisms in cell fate decision, i.e., **noise driven or signal driven** mechanism (**Coomer et al. (2022) Cell Systems 13:83-102**). Can the authors elaborate the relationship between their work and above two mechanisms?

Our analysis can be related to the work done on the quantitative Waddington landscape presented in (**Coomer et al. (2022) Cell Systems 13:83-102**). The regulative mode could translate into cell-cell interactions modifying the landscape, and would be the signal part. However, the mosaic mode, which we rephrased as lineage-dependent mode, as we defined, would have a larger definition than noise driven mechanism. As we understand, noise can drive differentiation of progenitors into different cell types, as shown in Coomer et al. Consistent with this, our toy model "lineage-only defined" diversifies the cells using a random walk with drift algorithm. However, another way to diversify cells is asymmetric division. In the specific case of C.elegans, given that the successive rounds of divisions and differentiations are invariant from one individual to another, we believe that is more asymmetric division than noise that drives the cell diversification (Gönczy, P. and Rose, L.S,Wormbook). We added a paragraph in the introduction to contextualize our study in relation with the papers Coomer et al. 2022, Li and Wang 2013 and Xue et al 2023.

2, The authors conclude that both modes (mosaic mode and regulative mode) could contribute to the development at various times in the various tissues. In the field of systems biology, it seems widely acknowledged that both noise and signal contribute to cell fate transitions (**Li and Wang** 2013 PLOS Comput Biol, **Xue et al**. eLife 12:RP88742 (2023)).

Indeed, it was expected that both signal (context dependent) and lineage dependent modes contribute. However, we propose a way to quantify these contributions without having to model a GRN unlike Li and Wang 2013 and Xue et al. 2023. This is made possible because we are using *distances between cells* instead of *individual cell expression profiles and spatial positions*. This approach complements the GRN based studies and can be used prior to them as a first inference approach before digging into more mechanistic considerations. We added a paragraph in the introduction and a paragraph in the Discussion to explain how our study related to the question of noise vs signal.

3, Usually, cell fate means cell type. i.e., similar cell fate (similar gene expression pattern in a high-dimensional gene expression space) should correspond to similar cell type. In current work, the authors show that **two cells with similar cell fate could have very different lineage** (Figure 5). Can the authors elaborate what this mean? Why is this observation reasonable? And what is the underlying mechanism for this?

Indeed, we observe that cells that are distantly related can lead to similar cell fate. This is consistent with previous biological observations such as the work of Sulston 1983 "The embryonic cell lineage of the nematode Caenorhabditis elegans", where they write for instance: "the embryonic lineage is highly invariant, as are the fates of the cells to which it gives rise. In spite of the fixed relationship between cell ancestry and cell fate, the correlation between them lacks much

obvious pattern. Thus, although most neurons arise from the embryonic ectoderm, some are produced by the mesoderm and a few are sisters to muscles”

The underlying mechanism could be that these cell fates are determined by the fact of ending up in similar contexts. Two cells have similar context if they receive the same signals from their neighbors. Signaling between neighbors is known to be at play in *C. elegans* in which for instance Notch signaling has been well documented and intervenes in different phases of development from very early interaction (responsible of symmetry break between ABa and ABp at 4 cell stage, Evans 1994) to induction of pharynx identity in descendants of both ABa and MS blastomeres (Kalb 1998, Mango 1994, Priess 1997). In our study, we don’t try to identify individually the molecules at play in each context but we treat the partial expression profile made of 266 transcription factors, as potentially relevant in defining the context of a cell.

4, Figure 5 shows that cell fate transition can go through different route for reaching similar endpoint. Similar conclusion has been predicted by **attractor landscape model based on gene regulatory networks** (Kang et al. Advanced Science, 8: 2003133 (2021).).

Since we don’t model the GRN, which would be large and very complex in the case of *C. elegans*, we don’t exclude nor validate the hypothesis that converging cell fates are due to different routes to reach the same attractor. An alternative hypothesis is that the cells, which are distantly related in lineage, are in different GRN, due to accumulation of noise and asymmetric divisions, and that it is in fact the context, i.e. the exposition to similar signals, that reunites them to the same fate.

5, There are grammar issues in the manuscript, e.g., line 271 “...we will explicit only one of them...”, line 404, “...with their context than with with their lineage inheritance...”. Please check the expressions throughout the manuscript.

Thank you for the careful reading. We corrected these mistakes in the revised manuscript.

Reviewer #3: This paper tackles the problem of understanding expression patterns on cell lineage trees. Here the authors develop metrics to explain expression differences between pairs of cells in terms of certain covariates, in particular lineage (or kinship) distance and context distance. They define context distance to be the correlation distance between the mean expression profile of the direct neighbors of each member of the cell pair.

For each cell pair, there is thus a lineage distance, an expression distance, a context distance, and a physical distance (measured in terms of the Delaunay graph). The study attempts to remove errors of confounding by stratifying the data and conditioning on certain distance types.

I think the paper presents an innovative and original methodology. I believe it to be an important addition to the toolset addressing how we analyze expression patterns in cell lineage trees. The biological insights - for example that lineage ("mosaic") influences are largely dominant, with

environmental ("regulative") influences sometimes appearing late - are helpful and in line with expectations for C. elegans.

We thank reviewer 3 for their careful reading and relevant suggestions, we provide below a point-by-point answer to their comments.

The problem of quantifying expression patterns on cell lineage trees has been receiving increased attention. Several workers have studied distances/differences between pairs of cells. Although the current manuscript goes further in defining distances in lineage, context, and space, as well as expression, I do think it would be helpful if the authors **place this work in the context of previous work** in this area, such as (though not exclusive to) the following: Lineage motifs as developmental modules for control of cell type proportions, **Martin Tran**, Ahmad Askari, Michael B. Elowitz, Developmental Cell 59, 812-826 (2024); Novel metrics reveal new structure and unappreciated heterogeneity in Caenorhabditis elegans development, **Gunalan Natesan**, Timothy Hamilton, Eric J. Deeds, Pavak K. Shah, PLOS Computational Biology, 19(12): e1011733 (2023); Maps of variability in cell lineage trees, **Hicks DG**, Speed TP, Yassin M, Russell SM, PLOS Computational Biology. 15(2):e1006745 (2019)

We thank the reviewer for very interesting literature. We have included these references in the introduction. While they are relevant to characterize the variability of some variable across multiple lineage trees, they do not directly address the question of characterizing the lineage versus spatial dependency of patterns of gene expression in a developing organism.

Below are some detailed points:

p.3, line 92: The effect sizes W_mosaic and W_regulative are introduced here well before they are defined. I suggest giving a short description of them and cross-referencing the Methods section where they are defined.

We added a short description on this header, pointing to the more developed definition below.

p.4, line 110: Please specify the unit of time used. If t=190 is about 6 hrs (line 112), this suggests 1 unit of time is about 2 mins? Given its importance in the discussion, **I would suggest the time be specified in hrs or minutes**.

Thank you for pointing out this mistake. 1 unit of time is 75 sec. We stated wrongly that the total duration is 6hrs. 6 hours was the full microscopy time that the authors reported in by Li et al, although none of their samples seems to have been tracked and processed until the end, given the number of time points https://dulab.genetics.ac.cn/data_sharing.html (uncompressed).The actual full duration is about 4 hrs. To avoid any doubt on the timing, we converted all the time steps into minutes in the manuscript.

p.4, line 138: Why is the spatial distance specified by the (discrete) steps in a Delaunay graph? I would have expected the Euclidean distance, which is continuous, to be a more accurate measure of proximity. I am not suggesting this be changed, it would just be helpful to have a comment about why this distance measure was chosen.

We used the number of steps in the Delaunay graph as a measure of spatial distance because we considered it more relevant than Euclidean distance with respect to the question of communication between cells. We hypothesize that signaling between cells occurs at membrane level (Guignard *et al.*, 2020). The Delaunay graph is a good proxy for cell-cell membrane contacts. Consequently, the interesting distance, when considering a pair of cells, measures how many intermediary cells lie between them, i.e. the number of steps in a Delaunay graph. We included an explanation of its interest in the Material & Methods.

p.5, line 161: The random walk model needs more explanation. x and v need to be defined.

Thank you for pointing this out. We have updated the Material and Methods section concerning the lineage-only model to make this part clearer.

Let x(t) the expression level of a given gene at time t. At each step this expression is updated in the following way: $x(t+1) = x(t) + v(t)$, where the component v(t) added to the current expression is built from its value at the previous step to which a random value is added $v(t) = v(t-1) + \epsilon$ where ϵ is drawn from a normal distribution. This is analogous to a random walk with drift performed by an agent with position x(t) and speed v(t). If there was not the random component $\epsilon(t)$, the agent would be diverging from its initial position at constant speed. The random part introduces a part of variability but overall, the expression over a cell cycle diverges. At each division, the first position of the daughters' cells are made to coincide with the last expression of the mother cells, so that the expression continues to diverge along each mother to daughter line.

p.5, line 177: The short cut phrase "...replacing inverting lineage and context" is unclear. Writing this statement out explicitly would be make the meaning more clear.

We have replaced this sentence by "replacing lineage with context, and reciprocally"

p.5, line 186: The context distances are stratified into 10 bins. Please be specific that (I assume) these 10 bins are created at each time point. I assume the bin sizes and bin positions vary over time as overall expression changes?

Yes, bins are created at each time points. We added this precision on the revised manuscript in the Material and Methods section.

p.6, line 198: Why do the definitions of W_mosaic and W_regulative involve subtracting the U statistic of the randomly-permuted model? **This does not appear to be a standard rank-biserial correlation so please provide justification or a reference**.

Thank you for noticing this. We clarified this point in the revised manuscript

Given two distributions, given all pairs of data points that are formed by one data point from one distribution and one data point from the second distribution, the standard rank-biserial correlation measures the proportion of pairs in which the data point from the first distribution is larger than the data point from the second distribution. In our case, the two distributions are respectively the distributions of stratified correlations derived from the expression data we are trying to characterize (either *C. elegans* data, or the lineage-only toy model), and the random null model which we take as reference.

Here is the derivation of our formula: the rank biserial correlation is often written as $\mathrm{rbc} = \frac{2\mathrm{U1}}{\mathrm{n1n2}} - 1 = 1 - \frac{2\mathrm{U2}}{\mathrm{n1n2}}$ (Cureton, E. E. (1956), Wendt, H. W. (1972)). Because $\mathrm{U1} + \mathrm{U2} = \mathrm{n1n2}$ (full calculation below*) it can also be written $\mathrm{rbc} = \frac{\mathrm{U1}-\mathrm{U2}}{\mathrm{n1n2}}$, which is the formula we adopt to define the weights W_lineage and W_context. Overall,

$$\mathrm{W} = \frac{\mathrm{U_{data}} - \mathrm{U_{random}}}{\mathrm{n_{data}n_{random}}}$$

*: $\mathrm{U1} = \mathrm{R1} - \frac{\mathrm{n1(n1+1)}}{2}$; $\mathrm{U2} = \mathrm{R2} - \frac{\mathrm{n2(n2+1)}}{2}$ where R1 and R2 are respectively the sums of ranks in sample 1 and in sample 2 after all data points from both samples are merged and ranked together. Thus, $\mathrm{R1} + \mathrm{R2} = \sum_{x=1}^{x=(\mathrm{n1+n2})} x = \frac{(\mathrm{n1+n2})(\mathrm{n1+n2+1})}{2}$. It follows that $\mathrm{U1} + \mathrm{U2} = \mathrm{n1n2}$.

p.6, line 224: When dividing the 3-dimensional space of lineage distance, physical distance, context distance into 8 regions, each region is termed a quadrant. This was confusing to me. I would suggest calling them **octants** since quadrants implies a division of a 2-dimensional space into 4 regions.

Thank you for the suggestion. We change "quadrants" into "octants" throughout the revised manuscript

p.8, line 271: The phrase "...we will explicitly only one of them..." needs to be clarified.

We have replaced by "Since these two measures are built the same way, we will explain in detail only one of them, the context dependency contribution"

p.8, line 274: In the sentence "We want to measure this strength independently of the lineage

distance which is likely to be a confounding factor in this correlation" I would suggest replacing "independently of" with "after controlling for".

Thank you for the suggestion, we have updated the text accordingly.

p.9, line 291: Reference is made to "W_regulative of the null model (by computing the U test between all pairs of time points within the null model)". How does this relate to the original definition that W_regulative ~ U_data-U_null?

Thank you for pointing this out. Indeed, a more detailed explanation was needed. We added a subsection in the Methods section in the revised manuscript.

p.10, line 338: "...between 10 and 50%". Doesn't the red line in Fig 3c only go up to ~30%?

Indeed, this is an error in the text. The red line only goes up to 30%. We corrected it in the manuscript.

p.10, line 367: Is "the percentage of cells in each configuration over time" actually a percentage of cell pairs? This refers to Fig 3c and its caption as well. I thought that the octants in Fig 3a were populated by points corresponding to cell pairs, not individual cells. I presume an individual cell could appear in multiple octants since it would usually be represented in multiple pairs.

Indeed, all the data points are cell pairs, and each cell belongs to n-1 pairs where n is the number of cells at a given time point. It appears clearly now in the revised Figure 3 legends and labels

p.11, line 403: "The new cells that just divided have adopted expressions that correlate more with their context than with their lineage inheritance." This sentence and the ones immediately after it were a bit confusing to me as it wasn't obvious that the context was causing the expression rather than the other way around. The later discussion about causality and time resolution was helpful but this paragraph could benefit from more explanation.

Thank you, we clarified this paragraph.

p.19, Fig. 3d: Does the fact that the red (context only) and orange (context and physical) lines almost overlap provide further evidence that physical proximity alone (without context) is not a major driver of expression?

Thank you for this interesting suggestion. To rephrase the idea, we see that the dynamic of the mean expression distance between cells as a function of time is similar if the cells are close in physical space or not (given that they have similar context). This suggests that physical proximity without context is not a major driver of expression. We've included this observation in the text lines (468-472).

References

Cureton, E. E. (1956). Rank-biserial correlation. *Psychometrika*, *21*(3), 287-290.

Wendt, H. W. (1972). Dealing with a common problem in social science: a simplified rank-biserial coefficient of correlation based on the U statistic. *European Journal of Social Psychology*, *2*(4)

Gönczy, P. and Rose, L.S. Asymmetric cell division and axis formation in the embryo (October 15, 2005), *WormBook*, ed. The *C. elegans* Research Community, WormBook, doi/10.1895/wormbook.1.30.1, http://www.wormbook.org
