# Supplementary material for: Assessing the relative contributions of mosaic and regulatory developmental modes from single-cell trajectories
Source: PLoS Comput Biol. 2025 Dec 15;21(12):e1012352. doi: 10.1371/journal.pcbi.1012352 (PMC12721551; doi:10.1371/journal.pcbi.1012352)
Supplement: Attachment — Submitted filename: response to reviewers.pdf [file pcbi.1012352.s008.pdf]

Paul Villoutreix
INSERM Chaire Professeur Junior
CENTURI Group Leader
Marseille Medical Genetics
Marseille, France

Dear Editors,

Please see the response to the reviewers' comments below. We have made significant revisions to the Introduction, Materials and Methods, and Results sections. We paid particular attention to clarifying our approach and included a discussion of the null models used in the paper.

Sincerely,

Paul Villoutreix and Solène Song

Reviewers' comments:

Reviewer #1: I appreciate the author's efforts in addressing our concerns. The revised version of the manuscript has indeed improved.

We thank the reviewer for their positive assessment of our revised document. We have addressed the typo and grammatical errors, as well as the remarks, see below.

Nevertheless, I still have some reservations about the article.

First, the language and grammar of the article should be thoroughly revised. Some typos and grammatically incorrect sentences include but are not limited to:
• Line 33: "One the remaining question is…" ✅
• Line 53: "Some studies other have focused on…" ✅
• Line 120: "…can be responsible of…" should be "can be responsible for" ✅
• Line 133: "…with reference to previously introduced random model" should be "with reference to the previously introduced random model" ✅
• Lines 304–307 could be written more formally. For example, instead of "vary a lot," one could write "are very variable." ✅
• Line 330: "the Pearson r" should be "the Pearson correlation r" ✅
• Line 340: "Material and Methods" should be "Materials and Methods" ✅
• Lines 449–450: "Figure 3 show" should be "Figure 3 shows" ✅
• Line 458: "…is similar if the cells are close in physical space or not" could be changed to "independently of their physical distance" ✅
• Line 483: "there is steep drop of the lineage" should be "there is a steep drop in the lineage" ✅

I appreciate the effort in including the other reviewers' suggestions to mention previous related work in the Introduction. However, I believe the Introduction needs further revision. For example, the authors argue in lines 41–42 that signal vs. noise quantification is not relevant to this study because "Here, in the case of C. elegans, which displays very stereotypical development with little variation, with invariant lineage, we expect little noise." At that point in the text, though, the authors have not yet introduced their main goal or clarified that they are focusing on C. elegans.

Thank you for pointing this out. We have re-ordered the introduction, splitting it into titled paragraphs. The objective of the work, a methodological development applied on *C. elegans* data, is now stated clearly at the end of the first paragraph of the Introduction : " *There remains no systematic method to quantitatively assess the relative contributions of these modes throughout the full course of development. Here, we present a method paper addressing this gap, applying our approach to* C. elegans *development"* **lines 28-31**

I still do not understand the dataset completely. In lines 148–149, the authors note, "At the last time point t=190 (about 4 hrs), there are 380 cells. By this stage, the embryos have completed all but the last round of cell divisions, and have produced 762 cells, for a total number of 1341 different cells observed across the entire development." Do the authors mean that embryo 13 has 380 cells tracked out of the 762 cells that an embryo would have at t=190, and that from t=0 to t=190, the dataset comprises a total of 1341 cell states?

At the final tracked time point, t=190 (approximately 4 hours), the embryo has completed all but its last round of cell division, resulting in 380 cells present at that moment. Cumulatively, from t=0 to t=190, the embryo has generated a total of 762 cells (the 380 cells at t=190, and all their ancestors). This implies that the lineage tree, as it stands at t=190, consists of 762 cell ids (including 380 leaves and 382 ancestor cells) over the total 1341 cells produced across the entire development. We reformulated this in the revised manuscript, **lines 153-158**

In lines 182–183 of the Materials and Methods section, the authors define the context distance between two cells A and B as the correlation distance between the mean expression profile of A's direct neighbors and the mean expression profile of B's direct neighbors. However, the neighbors of a cell are not defined until lines 314–315. It would be clearer if the authors reordered these explanations.

Aix*Marseille université | CENTURI TURING CENTRE FOR LIVING SYSTEMS | Marseille Medical Genetics | Inserm

Thank you for pointing this out. We added a line in the description of context distances to clarify this. "The neighbors NA and NB are the sets of cells which are one edge away in the Delaunay graph from respectively A and B" **lines 203-204**

In Figure 3C–E, the authors classify and describe various relationships between cells in the dataset. In particular, they focus on a subset called "context-only related" cells, which—according to line 437—is a key illustration of the regulative mode. They show in Figure 3D how the percentage of these context-only related cells changes over time, peaking between 62 and 190 minutes. However, Figure 2 indicates that the regulative mode is predominant at later times in all cell types, especially after 200 minutes. I am curious about how W_regulative relates to the percentage of context-only related cells. If my understanding is correct, a higher regulative contribution should correspond to a higher percentage of context-related cells. This would be consistent with observations in the Intestine—where cells are mostly clonally related, and the mosaic mode predominates. I would appreciate if the authors could explain these relationships further.

There is indeed a natural intuition that would suppose the following : the timepoints with high W_regulative should be associated with (i) higher number of cell pairs in the context-only related cells (as the reviewer infers) **AND** (ii) these pairs should have low expression distances.

It is worth noting that there are two limitations that might temper this intuition :

1. The regulative mode does necessarily operate predominantly in isolation. It is possible to have both a high value for W_regulative and a high value for W_mosaic. In that case, in contrast to scenarios where only the regulative mode operated predominantly, we don't expect a higher number of cell pairs in the context-only related cells and low expression distances.
2. Since W_regulative is derived from correlation of distances, pairs of cells with large context distances and large expression distances also contribute to a high W_regulative value. This is not captured by examining the context-only related octant as this octant only shows the low context distance pairs.

Despite these two limitations, we examined the relationship between W_regulative and the percentage of cells in the context-only related octant in the case of Skin late development. In this case, W_regulative is dominant from t=200 minutes onwards (with a low value of W_mosaic thereby reducing the impact of limitation 1).

We plotted in **Figure R1** (below, not included in the paper) the percentage of cells belonging to the population "context-only", at a given time point, versus the normalized mean expression distance (mean expression distance in the Skin population divided by the mean expression distance in the total pairs population). Data points are color-coded by time. Notably, for the time points highlighted by the reviewer, after t=200 minutes (red and dark red), the data points tend to cluster in the lower-left region of the plot. This indicates a low percentage of pairs that are context-only related. They do, however, have low expression distance. This low expression distance appears sufficient to account for high W_regulative values, without the need for a high context-only related percentage of pairs, contrary to the intuitive expectation.

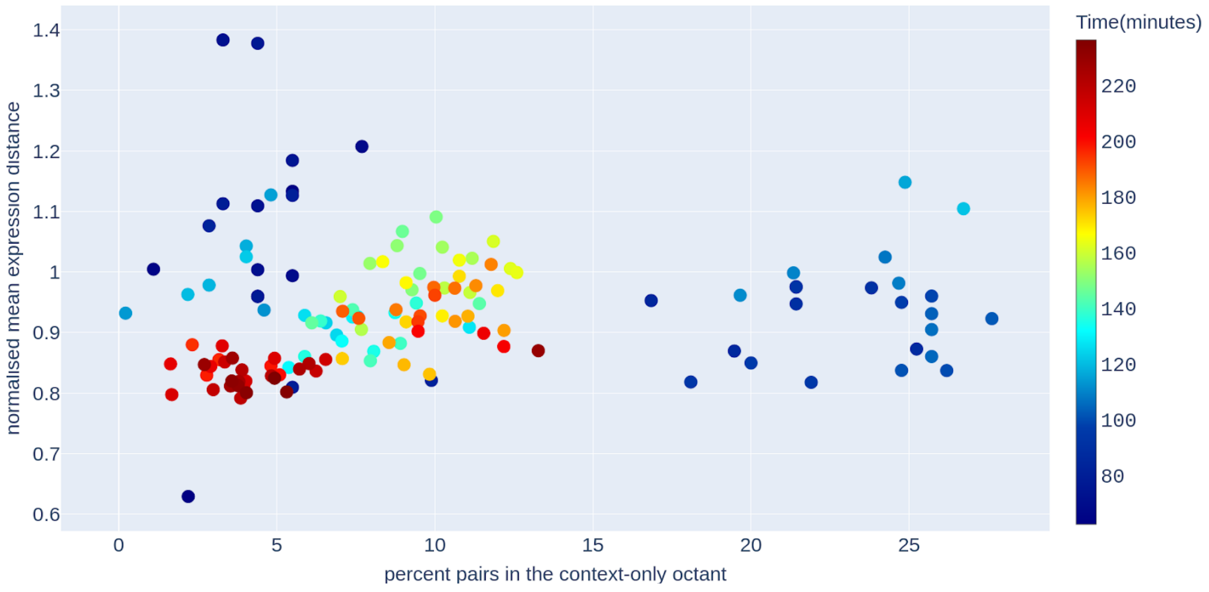

Figure R1 : Each dot represents a time point in the development of Skin tissue, with the time given by the colormap. This plot shows the normalised mean expression distances at this time point with respect to the proportion of cells in the context-only octant.

There are also early time points (blue) that appear in the bottom-right part of the plot. These points should, according to the previously stated intuition, reflect a purely regulative scenario, characterized by a high proportion of context-only pairs with low expression distances. Nonetheless, these time points are associated with low W_regulative. This discrepancy highlights the limitations of the intuitive expectation, likely due to the influence of limitation 1).

We decided not to include this additional figure in the manuscript but instead we added a paragraph clarifying that the octant analysis is complementary to the W_mosaic and W_regulative measures, and not a validation, **lines 447-455 :**

"*To summarize, we have defined two metrics, Wmosaic and Wregulative which quantify the degree to which the expression distances are correlated with respectively the lineage distances and the context distances. These metrics serve as proxies for the respective contributions of mosaic and regulative modes. However, they do not provide a completely disentangled and complementary weighting of both contributions, as they do not sum to a constant. Both metrics can be both simultaneously high or low. Also, they summarize all the cell pairs at a given time point into a single value. We complement these metrics with an explicit, joint analysis of all four distances: lineage, context, spatial network, and expression, to highlight specific populations of cell pairs of interest.*"

Regarding Figure S3, the percentage of clonally related cells in the Intestine is very high but sometimes drops to 50%. I am wondering what types of cells make up the remaining 50%.

Thank you for pointing this out.

The remaining 50% is made of cells that are in so-called "unoccupied" octants. Indeed, these unoccupied octants which are pairs of cells (i)close in lineage and space but far in context (ii)close in lineage and context but not space, and (iii)close only in lineage: , when pooling over all pairs in all tissues, they concentrate only about 0.3 and 0.7 % of pairs of cells. But when analysing separate tissues with small numbers of cells, they represent a non negligible proportion of the pairs of the tissues, which is the case for Int and Mus. We add here a plot in **Figure R2** for the raw number of pairs in the "unoccupied" octants, which transiently occupy these octants because of the sharp threshold between being considered close or far.

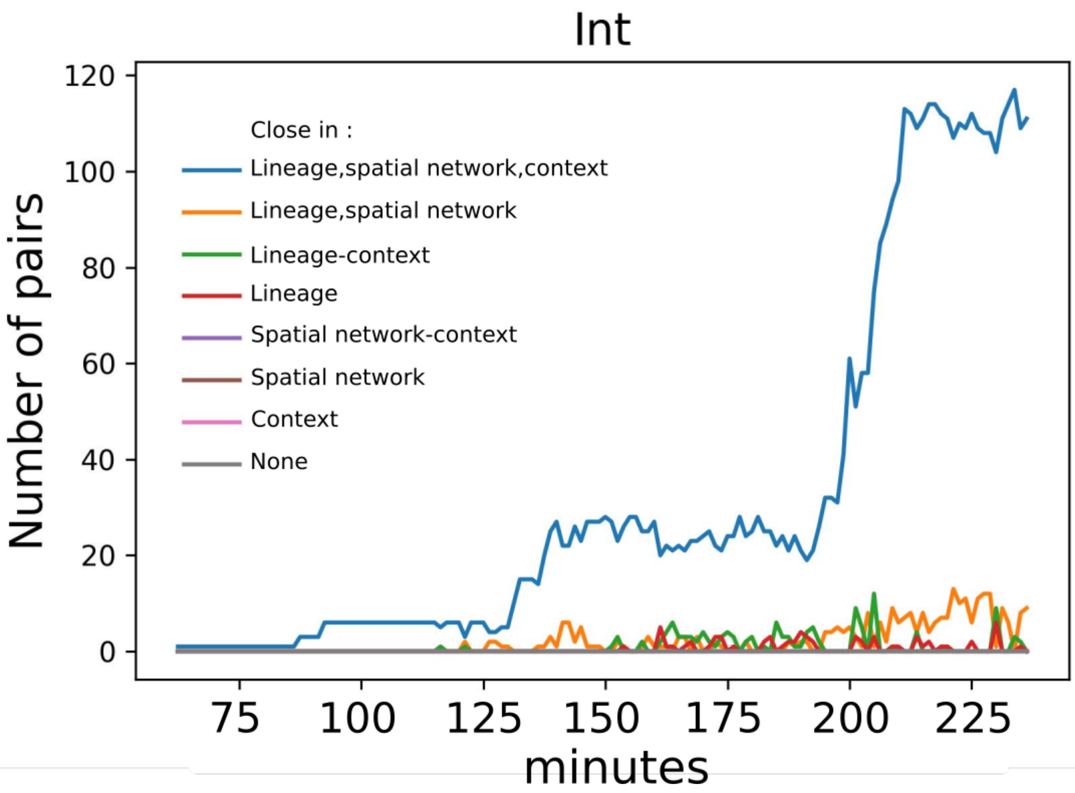

Figure R2 : The raw number of pairs of cells in each of the 8 octant. These 8 octants are the 5 that are considered in the main figures, and additional 3 octants, considered as globally unoccupied when pooling all the cells, of pairs that are (i)close in lineage and space but not in context (orange) (ii)close in lineage and context but not in space(green) and (iii)Close only in lineage

Reviewer #3: I accept the responses and manuscript changes and recommend publication.

We thank the reviewer for their positive evaluation of our revised manuscript.

I do have a comment regarding the "Lineage-defined model" on page 6/23 (thank you for explaining it in more detail). I think there is wide scope for defining null models that exhibit purely mosaic variation. The Langevin-type equation the authors have chosen is a specific type of random walk on the lineage tree that has limited memory. One could imagine null models with longer-term memory that explain more of the correlations at large lineage distances. Do the authors think this could affect their conclusions? If so, I recommend the authors note this in the text by saying that the test for significance (for mosaic variation) is sensitive to the specifics of the lineage-based null model.

Thank you for giving us the opportunity to put in context our model compared to other stochastic processes.

We tried to propose a simple model of differentiation trajectories. Since we want the cells to be differentiating, the expressions must overall diverge progressively and consistently (with reduced chances that the modeled expression returns to previous values). This means that the walk that defines the expression levels must have, indeed, as the reviewer points out, a long-term memory. This cannot be achieved with a pure brownian motion. The equation we used is adapted from the core of the PROSSTT algorithm (Papadopoulos et al., 2019, Bioinformatics), which we named erroneously a “random walk with drift” when it is actually a Random Acceleration Process (RAP). The RAP follows an undamped Langevin equation, that can be written as d2xdt2 = where is white noise. In this setting, we have the relationship x(t+dt) = x(t) + v(t)dt, where v(t) is a brownian motion, v(t+dt) = v(t) +(t)dt, and x(t) is the integral of this brownian motion. v(t) has a short term memory as a markovian process

which depends only on its previous value, while x(t) is the cumulative sum of a markovian walk, therefore has long term memory. Specifically, if v(t) has a positive trend over some period, x(t) will persistently increase. Thus it is very unlikely that x(t) returns to previous values. In that sense, **the RAP has a long term memory**.

We agree with the reviewer that one can imagine other methods to achieve a long term memory expression model. The simplest being to keep the velocity constant. Then we could also have adopted other non-markovian processes such as fractional brownian motion which include correlations between the increment steps (brownian motion is a specific case for H = ½)

We rewrote the lineage-defined model paragraph in the Methods section to clarify the model's formalism. And we acknowledged in the Results section **(lines 410-417)** that other models, more complex or noisy, could have been used and that this part of the validation of our metric depends on the choice of such model :

"This specific test is sensitive to the model we chose for the lineage-defined expression. We opted here for a model in which cells persistently diverge from their initial expression and from each other during their cell cycle. At the end of the cell cycles, two sister cells are different in expression. However, since they inherit their starting expression from their common mother, they are more alike than distantly related cells. While alternative models, more complex or noisier, could have been used, the lineage-defined model used here relies on simple assumptions, making it highly adapted for our study."

Reviewer #4:

Recent progresses in the study of embryonic development of various organisms make it possible to quantify development at increasing temporal and spatial resolution in 3-dimensions. Quantitative measurements of both geometry and genetic activity in 4-D of each cell are available within the cell network of the whole embryo. This new possibility to construct quantitative atlases of development offers completely new opportunities to study how cells progressively acquire their identities in the mature tissues. In particular, if it is clear that the two main modalities mentioned by the authors, namely Mosaic and Regulative, contribute to cell fate acquisition, how much each of them is involved at different stages of the development, and in different parts of the embryo, remains poorly understood.

The paper presents an original method to assess the relative importance of these Mosaic and Regulative modes at various stages of the embryo development. Contrary to previous methods for which a specific choice is usually made to quantify the distance between cells (e.g. distance between cell expressions or between cell lineage trees), this method uses a comparative analysis of several cell to cell distances, reflecting either topological, lineage or gene expression distances between cells. The aim is to analyse how these distances gets correlated (or not) during the development. The authors use C. elegans as a model organism to test and illustrate their method. Its stereotypical development with little noise makes it well adapted to map single cell expressions data to imaged cells in both space and time (that was done in a previously published dataset).

Main assessment

Overall the proposed method is original and looks sound. It offers a new and interesting perspective to disentangle the relative contributions of cellular context and cell inheritance in the acquisition of cell fate throughout embryo development, based on distance correlations. It is shown that either mosaic or regulative mode may dominate in different tissues depending on the development stage and that very different cell trajectories, both in lineage and space, can converge to similar fates at very distinct places in the embryo (reminiscent of convergence phenomena in evolution trees).

After reading the whole paper, one can perhaps regret that the method does not clearly highlight new biological findings on the development of C. elegans or provides insights on some fate acquisition mechanisms. But this may be due to the early stage of use of this method, and further research should probably be made to reach such new biological insights. In the meantime, this new method clearly deserves to be published, and this paper can, in my opinion, be kept **mainly a concept/method paper (provided clearly stated).**

Thank you for pointing this out. We have stated explicitly that the objective of the work is a methodological development applied on C.elegans data at the end of the first paragraph of the Introduction : " *There remains no systematic method to quantitatively assess the relative contributions of these modes throughout the full course of development. Here, we present a method paper addressing this gap, applying our approach to C.elegans development"* **lines 28-31**

Detailed comments

- The notion of cell identity and cell fate are not defined. As they are the central concepts on which the paper's method is based, I think they should be thoroughly defined in the introduction.

We have defined that cell “state” is often measured through the transcriptomic profiles, and we kept “fate” for the final cells state, discarding “identity” to reduce the number of synonyms, **lines 2-5**

- The Figure 1.A in this respect could be improved. Why are fates represented completely differently (colored dots / colored cells) in Mosaic and Regulative modes ? a unified description would convey more the general idea of comparing distances.

Thank you for the suggestion. We have modified Figure 1A so to keep the colored dots as representation of cell fate.

- p3 l53: Some studies other --> Some other studies

Thank you for pointing this mistake out. We corrected this

- There are a certain number of imprecisions/errors in the formalization. I would recommend to make a thorough pass on the method part to improve clarity. For example:
- W is defined as a "contribution" (bot p 4). But it is not clear what (mathematical) object is a contribution: a real ? a vector ? a series of real weights ? …
Thank you for pointing this out. We stated clearly that
“*We introduce a quantification method to assess the respective contributions of the mosaic and regulative modes in C. elegans development which we denote by Wmosaic and Wregulative and detail below. These are coefficients with values comprised between −1 and 1 that we obtain for each time point, for either all the cells in the embryo or only those which participate in a given tissue*”
lines 128-132

- information on a cell: position in the 3D-space. Do you mean position in the graph representing the embryo + the 3D-coordinates of the cell in space ? The 3D coordinates may vary between two embryos (if the two embryos do not have the same size, or some cells have a slightly different geometry). In principle 3D coordinates are not invariants. How the combination depends on different dataset geometries ? The method should be thought to be robust to this type of variations. Should you consider "normalized 3D coordinates" at least in principle, or take relative 3D positions of cells with respect to one another, other ? This should be discussed at least.

We actually never use the 3D coordinates directly in our computations but we always use the distances as shortest paths in the Delaunay graph, which we made more clear in the revised manuscript, by replacing “spatial distances” by “spatial network distances”. This measure should not be sensitive to embryos sizes nor overall densities. To check this, we computed violin plots of the distributions of Delaunay distances in all the embryos of the dataset at 3 different timesteps (time step 50, 100, and 150) and we observe very similar distributions across embryos for the same time point, except from the latest time point (step 150, about 187 min) in which some of the embryos tend to have higher distances than others, maybe due to some desynchronisation.

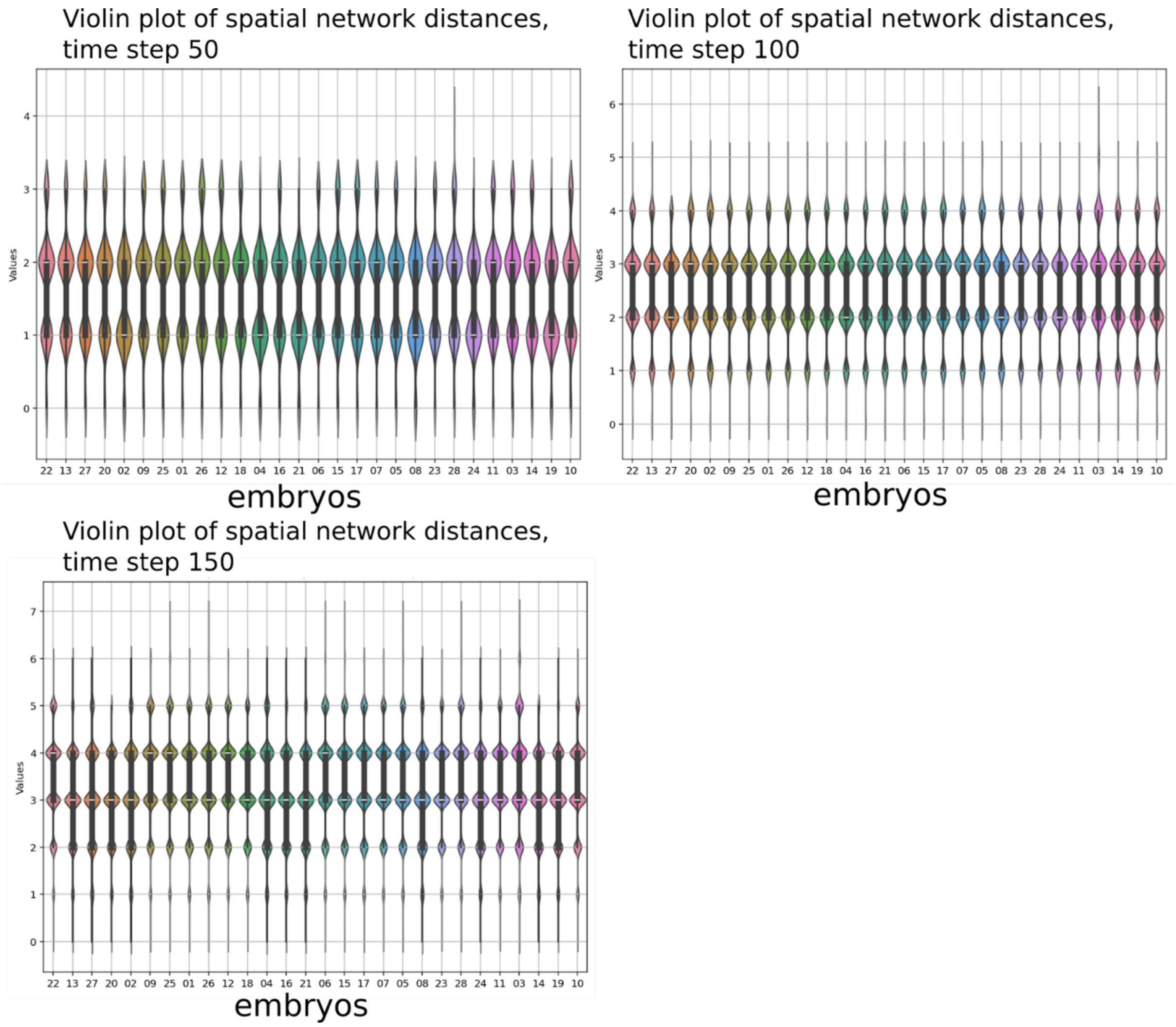

Figure R3: Comparison of the distributions of spatial network distances between embryos of the dataset, at 3 different time steps.

- In the section "Distances" p 5, from a conceptual point of view, it is not obvious why you are defining the distances the way you do. One would expect that each distance paragraph starts by a description of the notion that the authors want to define from a biological point of view (no math, no implementation details). Then this could be followed by an explanation of how this notion is well quantified by the chosen method.

Thank you for the suggestion, we clarified the intuition behind the various distances in each of the distance paragraphs in the method section.

- In particular, I was a bit confused by the second artificial model (lineage-defined) supposed to present an "ideal case of a Mosaic development" (p6 l191). It is not obvious that the lineage distance reflects well Mosaic development. ***The intuition behind this statement should be explained in more detail and discussed.*** Figure 1.A (left) actually illustrates this issue: Although two daughter cells of the top mother cell have a minimal lineage distance (they share the same mother cell with five colors), they obviously inherit different amounts and types of fate potential from their mother cell. Hence while the two daughter cells have a small lineage distance, they inherit different "expressions" from her. As this expression distance can be made arbitrary large, it is not obvious in general why should lineage distance correlate well with expression distance in the Mosaic mode.

We assume that in mosaic development, the daughter cells inherit a large portion of their expression symmetrically from their common mother cell, and another portion asymmetrically, due to asymmetric distribution of factors (transcripts, proteins, epigenetic marks, etc).

In Figure1.A, the circles represent the potential fates which are still accessible to the cells. One could see them as representing the asymmetric factors only.

In the lineage-defined artificial model, the symmetric inheritance is conveyed by the fact that upon division, the first time point expression of both daughter cells are the same as the last time point of their mother. Whereas the asymmetric part is conveyed by the fact that during the cell cycle of a cell, the expression diverges progressively from this initial expression. This divergence is simulated by a random accelerated process, which also leads both daughter cells apart (see answer to Reviewer #3).

- The description of the distances could be improved on the form. One can guess the missing information, but I had to read twice this part to make sure that what I understood was indeed what was meant. In each distance definition, the notation d(A,B) should be used for two cells A and B considered in the embryo at the same time t.

We defined better the distances, with the same notation d(A,B). Thank you for the suggestion.

Please choose network, graph or tree for the lineage tree (but not all these terms interchangeably). You do not need to say that edges are not oriented (in a lineage tree they must be, or alternatively you need to define a single node as the root node (the fertilzed egg) and explicitly say it).

Thank you for pointing this out. We removed “graph” from the manuscript, and kept consistently “tree” to refer to the lineage tree.
The only occurrence of “network” remaining, when discussing the lineage tree, is now in the Material and Methods section when defining lineage distances, to clarify that we treat the lineage tree as a network. Specifically, the distance defined as the shortest path in the network derived from the cell lineage tree do not require edges to be oriented, nor a specific root node.

We consider a tree as a particular type of network, which makes the use of both terms redundant. We kept this occurrence of the word network to make the link between “lineage tree”, understood as the biological hierarchy of cell divisions, and the underlying network where cells are nodes and ascending/descending relationships are edges.

Neither u, v nor <u> in the definition of expression distance are defined. There is a typo in the denominator of the formula: the minus sign between the two norms should be removed.

Thank you for pointing out this mistake. We corrected it, and we defined the average values everywhere.

The spatial distance could be better named as this evokes a euclidean (distance between cells in space). It could be renamed network distance.

We replaced everywhere by “spatial network distance”

"Number of steps between two
cells": steps is not clear. This should probably be replaced by "number of edges in the Delaunay graph". You could also explain that edges in the Delaunay graph correspond to cell to cell interfaces.

We rewrote the definition of spatial network distance in the Material and Methods section to clarify this. Thank you for the suggestion.

In the context distance, \bar{v_A} is not defined. Notation here should be detailed with care as two levels of means are taken, which might be confusing.

We changed to <v_A>, consistently with the previous formula of correlation distance, and specified its meaning

- It is not usual to mix description of the computational implementation (used primitives in scipy) with the formal description of the distances. You should probably postpone these implementation details in a small special dedicated section.

We removed the implementation details, they are already in the code. We focused on formal description of the distances in the text.

- A figure showing the expression profiles (page 5) and the tissue annotation would help greatly understand the geometric/lineage structure of the worm.

Thank you for the suggestion. We added Figure supplementary 6 to show the cell types spatial distributions and an UMAP to visualize the expression profiles, with terminal cells colored according to tissue label. The lineage tree is very large, so we did not include it. The full tree and terminal fates can be found elsewhere (Ma et al., 2021, Nature Methods).

- In the Lineage-defined model, in x(t+1), it is not clear what means 1 and what are the units of time. This should be explicitly explained. In particular, due to this the random walk description appears to be weird by summing a position and a speed (x(t+1) = x(t) + v(t)). This would be cleaner and more sound to write: x(t+ Dt) = x(t) + v(t).Dt so that one sees properly matching units. epsilon should also be consistently epsilon(t) everywhere.

Thank you. We extended the description of the lineage-defined model and clarified the various notation. See answer to Reviewer #3.

- p8 l302, what is the different between "physical distance" and "spatial distance" ? Again (if I understand correctly), please consider using one term, and not these ones that refer to euclidean distance in the 3D space, e.g. like "network" or "interface" distance.

We now call this distance consistently “spatial network distance”. Thank you for pointing this out

- p9 l336 I did not understand "6 relationships".
We reformulated the introduction of the Results so that this sentence is no longer present. This paragraph has been replaced by:

“The simplest way to quantify these correlations is to calculate the Pearson correlation r between the expression distances and, respectively, the lineage distances and the context distances. However, one can immediately see as shown in Figure 1B and S1 Fig that these relationships are intertwined through confounding effects. For example, because cells which just divided are still close to each other, it is expected that two cells close in lineage, which just branched off from a common ancestor are still close in space [34]. Because they are close in space, they would share a number of common neighbors, resulting in a small context distance too.” **lines 363-375**

- p9 l354-359. This is a unnecessary repetition.

We removed the useless repetition of the idea of the method “ A natural way for characterizing the context dependency contribution is to evaluate the strength of the correlation between context distances (the factor we assess) and the expression distances (the read-out).”; keeping however the technical steps that allow to interpret Figure 2. **Lines 383-403**

- p10-11 While this paragraph presents a very interesting analysis of fates based on extreme values of the distances, its title (l417) is quite unclear. Titles that would present the method rather than results could also be considered.

Thank you for pointing this out. We simplified this title into “Clonally-related cell pairs exhibit similar expression profiles” **line 446**

- p11 l450, "Figure 3 show" add an s

Thank you, we corrected this typo.

- p 12 l509, "a particular pair of cells that belong" add an s

Thank you for noting this typo.That this particular part does not exist in the revised manuscript due to reformulation of the sentence into *“In the previous sections, we showed that some pairs of cells share similar context and expression profiles despite being distant in both lineage and spatial networks: the cell pairs in the red octant in Figure 3. We now focus on one such pair observed at the final time point (237 min 30 sec) in Figure 5: ABplaapppp (green) and Cpappd (red).”* **lines 532-536**
