# Supplementary material for: Assessing the relative contributions of mosaic and regulatory developmental modes from single-cell trajectories
Source: PLoS Comput Biol. 2025 Dec 15;21(12):e1012352. doi: 10.1371/journal.pcbi.1012352 (PMC12721551; doi:10.1371/journal.pcbi.1012352)
Supplement: Attachment — Submitted filename: Response to the reviewers.pdf [file pcbi.1012352.s009.pdf]

Paul Villoutreix
Junior Professor Chair INSERM
Marseille Medical Genetics
Turing Center for Living Systems
Aix Marseille University
Marseille, France
paul.villoutreix@univ-amu.fr

Dear Editors,

We are very happy to share our revised manuscript. We have substantially edited the manuscript to improve clarity, rigor and precision. Specifically, we have worked on the *introduction* to improve the logical flow. We have clarified the *Material and Methods* section, in particular the mathematical statements, but also the transitions to guide the readers through the various sections. Finally, we have been careful to separate the methods from the results, and have reworked the *Results* section for clarity.

Beyond these clarifications, our conclusions remain consistent with those of the previous version. We anticipate that these improvements will ease readability for readers with either biological or mathematical background.

You will find below our answers to the reviewers.

Thank you very much for considering our revised manuscript for publication in *PLoS Computational Biology.*

Sincerely,

Paul Villoutreix
Solène Song

Reviewer #1: I appreciate the authors' efforts in revising the manuscript and responding to my questions. I accept the responses and the revisions and recommend the manuscript for publication.

We thank the reviewer #1 for their positive assessment of our manuscript.

Reviewer #4: I do appreciate the efforts of the authors to answer my previous comments. However, I still think that the method description needs clarification: for the moment, part of the method text is still quite difficult to read (the formalisation lacks rigor and precision), and I would not be able to reproduce the authors method independently as the description still contains ambiguities and notational issues that blurs the overall approach (which otherwise remains original and interesting).

We thank the reviewer #4 for their suggestions which helped us improve the manuscript. Based on their comments, we have substantially edited the text to improve readability and clarity, and to clarify our core assumption and its limitations.

Here are comments to help identifying and addressing these issues (they are mainly related to the method section).

p2, L6-7: subtitles in the introduction are not usual. At least the first title is very strange and poorly introduced by the two first sentences. it sorts of restates the title of the paper slightly differently.

We removed the subtitles, and we re-organized the introduction to improve clarity and logical flow.

p 3, L70-74: About the core assumption: the closer the cells in the lineage tree, the higher their expression profile.

In a Mosaic-based embryo lineage tree that develops two symmetric sides (Left-Right), two cells with identical fates taken respectively in different sides Left and Right of the embryo, will be far in the lineage tree (their closest common ancestor is close to the zygote cell). However, their expression neighborhood will be similar.

This is thus a situation where in the Mosaic development, two cells have a high lineage distance and same expression. However, according to the core assumption of the paper, they should have distant expression profile, which is obviously not the case.

Should the assumption of the authors remove first symmetries in the embryo ? (i.e. their assumption holds if L/R symmetry is discarded) ? Are these situations rare, when correlations are considered ? I think the author should develop the intuition behind their core assumption and discuss these questions to justify their choice.

Thank you for questioning our core assumption: under a predominantly mosaic mode, cells that are closely related in the lineage tree are expected to exhibit higher similarity in their protein expression profiles. We have added a paragraph in the introduction (copied below) to clarify it and highlight its limitations. We assume that, despite identified cases where the assumption does not hold, it applies to the majority of cell pairs, especially when a great number of protein expressions are considered.

In the specific case of the left-right symmetric cells, we agree that the lineage distances should not correlate with expression distances. This could reflect either regulative influence, or mosaic influence that fall outside the scope of our core assumption. If the latter applies, we would argue, as the reviewer anticipated, that it concerns only a small subset of cell pairs. Indeed, let N the number of cells at a given time point. If all cells could be left-right paired, we have N/2 left-right pairs of cells for a total of N(N-1)/2 pairs. At the start of our analysis, there are 24 cells, thus 18% of pairs that are left-right symmetric. This percentage decays very fast (decays to 7% at 38 cells, until 0.0007% for the maximal number of 380 cells). Moreover, in C.elegans, not all cells can be left-right paired. There is a list of fully differentiated cells (https://www.wormatlas.org/celllistsulston.htm) in which we can find all the functionally left-right symmetric pairs of cells, and this boils down to 48 pairs.

The added paragraph in the Introduction **(lines 56-78)**:

“The central assumption is that, under a predominantly mosaic mode, cells that are closely related in the lineage tree are expected to exhibit higher similarity in their expression profiles. Conversely, under a predominantly regulative mode, cells with similar contexts, regardless of their lineage relatedness, should display greater similarity in expression profiles. The use of the correlation between lineage distance and expression distance as a proxy for mosaic mode rests on a simplifying assumption: during each cell division, most molecular information from the mother cell is transmitted to both daughter cells. As a result, their expression profiles remain close to that of the mother and, consequently, more similar to each other than to those of more distantly related cells. The differences observed between sister cells are expected to include a smaller asymmetric component, either inherited or acquired during their life cycles, that drives their gradual divergence.
There are specific situations in which this assumption does not seem to apply, at least for a part of the inherited information. For example, during the early divisions of C. elegans, the asymmetric segregation of P-granules specifies the germ line [16] [17] [18], leading to an abrupt fate asymmetry. Lineage-relatedness and expression similarity also clearly diverge for left-right symmetric cells, which can be distantly related in the lineage yet exhibit highly similar expression profiles. The latter case may reflect either regulative processes or lineage-dependent inheritance mechanisms that fall outside the scope of our simplifying assumption. Nevertheless, our assumption remains practical, simple, and hopefully broadly applicable, since we expect it to hold for most cell pairs during development, and particularly when large sets of gene expression profiles are considered.”

p4 L115-188: the notion of "weight of factors" is not defined and is used intuitively. At this point, without having read the rest of the paper, it is very unclear. The weight in which measurement process ? which factors ? are we still referring to the distances that are introduced before ?

We removed the term “weight” and now consistently refer to the “contributions of mosaic and regulative modes” to reduce ambiguity. Thank you for highlighting the previous lack of precision and the mix between intuitive concepts and quantitative measurements. We hope the revised paragraph in the Introduction (copied below) now clearly links the intuitive notion of “respective contributions” to their formal, quantitative definition.

Added paragraph in the introduction **(lines 79-89):**

“We developed quantitative measures that serve as proxies for how much information is passed on through lineage-inherited factors versus interactions with neighbors, which we refer to as the contribution of the mosaic mode Wmosaic and the contribution of the regulative mode Wregulative, respectively. More formally, as detailed in the Materials and Methods section, Wmosaic is built from stratified correlations [19] between lineage distance and expression distance for all cell pairs at all time points. For a given time point, the distribution of these stratified correlations is compared with a random expression model, and Wmosaic represents the difference between the real C. elegans data and the random expression model. Wregulative is defined analogously, but replacing lineage distance with context distance. These two metrics, while they do not imply direct causality, can suggest the presence of underlying biological mechanisms [20].”

p4: First paragraph. Why are Wmosaic and Wregulative values between -1 and 1 ? This should be explained as one naturally expects these W to be between 0 and 1: 0 meaning no contribution at all, 1 meaning full contribution. I understand latter in the text, that these factors are linked with some notion of correlation (which is then properly defined even farther in the text). I strongly advise the authors to consider the order in which they introduce concepts and definitions. This order should be so that one does not need to read the rest of the text to understand a definition of a statement. Please check this issue, not only in this particular instance, but throughout the method text. The precision of all these definitions is key and should be made at the usual scientific standards: for the moment, and even after the revision, although I am familiar with these types of formalizations, I am really struggling to understand clearly what was precisely done from the description in the text. See also below.

Thank you for this constructive comment, which reminds us that the Methods section will be read before Results and that all terms must be clearly introduced there. We wrote a detailed introduction of the Methods section that walks the reader through the logic of our metrics, and the different subsections (lines 99-123). Additionally, the information that W_{mosaic} and W_{regulative} range from –1 to 1 has been moved to the subsection on the calculation of W_{mosaic} **(line 301)**, where it can be presented in the proper context.

Added detailed introduction of the Materials and Methods section **(lines 99-123):**

"We introduce a method to quantify the respective contributions of mosaic and regulative modes in C. elegans development, denoted Wmosaic and Wregulative. An intuitive explanation of these measures is provided in the Introduction and their detailed construction is described in the subsection Calculations of the contributions Wmosaic and Wregulative, following the presentation of all required inputs.

This method can be applied only when lineage relationships, spatial positions, and temporal expression profiles of cells are available, as is the case for C. elegans (see Datasets subsection). Using this information, we define four distance measures: (1) lineage distance, (2) spatial network distance, (3) expression distance and (4) context distance. The precise definitions of these distances are provided in the Distances subsection.

The construction of Wmosaic and Wregulative requires a random null model used to generate reference statistical distributions (described below). To validate our metrics on a simple and extreme case, we designed a model that represents purely lineage-defined expression profiles, allowing us to test whether under this model Wmosaic is high and Wregulative is low as expected. The random null model and lineage-defined model are described in detail in the Artificial models subsection.

In addition, we introduce the definition of octants, which allows us to identify pairs of cells that are expected to undergo exclusive regulative development, disentangled from mosaic influences, namely those that share similar expression profiles and similar neighbors' expression profiles, yet are distant with respect to the lineage distance and the spatial network distance.

Finally, we introduce how to compute the rearrangement rate, a process that affects the spatial organization of cells and consequently the contribution of the regulative mode Wregulative."

p5: L141-158: The datasets section does not mention which embryos where used. Most of the related information is badly placed in the next section (spatial position). Is it 1 or several embryos that were used in this study ? According to L152, I understand that this is one (embryo 13). This should be in the dataset section.

The datasets section is organized into two subsections: spatial positions and expression profiles. We clarify that only one embryo is used for the spatial positions, whereas the expression profiles are combined from hundreds of embryos using reporter lines (Ma et al.). To make this clear since the beginning of the Datasets section, we added the following information **(lines 129-136)**:

"We combine two published datasets to obtain spatial and expression information:

• cell positions in 3D space, obtained from one embryo among 28 of the datasets of

Li et al. [3], and

• protein expression profiles from Ma et al. [10], who combined reporter-line

measurements from hundreds of embryos to reconstruct expression dynamics for

all cells throughout the lineage. The expression profiles are partial because the authors restricted their measures to a curated subset of transcription factors likely to be relevant for development."

p5: L145: "partial expression profile": please, explain in which way this is partial. Is partiality an issue at all for the conclusions ? why ? This should be discussed

Ma et al. [12] combined reporter-line measurements from **hundreds of embryos** to reconstruct expression dynamics for all cells throughout the lineage. The expression profiles are partial because the authors restricted their measures to a curated subset of transcription factors likely to be relevant for development.

Thank you for pointing this lack of clarity in the Methods lead paragraph, we added this precision in the manuscript **(lines 134-136):**

“The expression profiles are partial because the authors restricted their measures to a curated subset of transcription factors likely to be relevant for development.”

p5: L177: "network" should probably be replaced by "tree-graph", more standard and adequate in this context

We changed “network” with “tree-graph”

p6: L181: According to its name, the function ShortestPath() returns a path, not a length. I therefore suggest to rewrite "d(A,B) = ShortestPath(A,B)" as d(A,B) = length(ShortestPath(A,B)). Same at line 193.

Thank you for the suggestion, we changed the notation.

p6: L184: you should mention explicitly that x_A and x_B are vectors of reals with a certain dimension, and which one?.

We added this precision : xA and xB are vectors of dimension M=266 (number of genes measured), representing the expression profiles of cells A and B

The notation for mean value should be defined here (this is another instance of the ordering problem mentioned above). This should mention the set over which the mean value is taken: for the moment, I don't understand precisely what is : is it an average over all the "same cells" in a set of embryos ? Or is it an average over a specific tissue type in the same embryo ? I bet this is the second, but please realize that this is not defined at all in your definitions and that this is critically lacking at this moment of the text. Many things like this one are left not precisely defined to follow the text without ambiguity.

The notations were indeed ambiguous. We have revised the notations to distinguish vectors using boldface, and to explicitly write the averaging operation whenever it is applied. This clarifies the distinction between averaging over the elements of a single vector (yields a scalar value), and averaging multiple vectors to produce an average vector **(lines 176-179, and 197-200)**. In the case <xA> and <xB> (noted \bar{xA}, \bar{xB} in the revised manuscript), they are actually scalar values, as they are the mean over all elements in vectors xA and xB, respectively. We hope we wrote this more clearly in the revised manuscript.

p6: Spatial network distance: if I understand correctly, the spatial network distance is defined by the a number of edge in the cell network of the embryo. If this is correct, I strongly suggest not to use the term spatial, that usually refers to some euclidean or at least metric-based embedding space (spatial would mean here that you intend to use cell geometry or coordinates). What you use here is usually called a topological distance (just using the adjacency connections in the network). The whole paragraph spatial network distance is ambiguous in this respect (for instance why are you speaking of cell spatial positions line 198-194 if they are not used ?). Consider renaming this distance (spatial is not adequate), e.g. network topological distance, or embryo cell-to-cell topological distance (to be completely explicit).

The cell spatial positions are used in the construction of the spatial network. Indeed, we approximate cell-cell connectivity (in absence of membrane staining) by constructing a Delaunay graph in which vertices are placed at the spatial positions of the individual cells.

We clarified this in the Material and Methods **(lines183-192)**. It is important to us to leave the word “spatial” in the name “spatial network distance” because this network does reflect spatial proximity, which appears indeed unclear in the former manuscript.

p6: The notations "< x_A >" (e.g. line 184, 205) and "< x_i>_{i in N_A}" (line 206, 207), are different: in one notation one can see the set over which the index varies and in the other, one doesn't see. This type of variation in the notation of the same mathematical notion is confusing.

Thank you for pointing out this lack of precision. We explicitly wrote the expression of each mean and harmonized the notations **(lines 178, and lines 198-199).**

Indeed, there was an ambiguity around the mean over the elements of a vector (a scalar), and the mean expression profile over all the expression profiles of the neighbours of a given cell, which is itself a vector (which we denote \mu_A, \mu_B in the revised manuscript). Sorry for the confusion, we hope there is no ambiguity left after the modifications.

p6: I would strongly advocate that you consider a careful, precise and unambiguous rewriting of this entire section (starting just after the title "Materials and methods" and finishing just before "Calculation of the contributions Wmosaic and Wregulative") and present the ideas in a more streamlined way. I would also advise the authors to have it proof-checked by some expert with mathematical background in this domain.

Thank you for the suggestion, we added a lead paragraph that walks the reader through the different subsections **(lines 99-123)**. We proof checked the mathematical notations.

p7 L241: "dt = 1": what is the unit ? in particular with respect to a cell cycle that you mention several times in this paragraph.

Indeed, the previous use of dt, appropriate for continuous processes, was unnecessary and unclear. We removed it and replaced it with a discrete notation x(i+1)=... and stated clearly that each cell cycle is divided into three steps **(lines 228-229).**

p7 L247: if epsilon(t) is drawn from a normal distribution, it means that the maximum value of epsilon(t) is less than 1. This in turn means that the maximal rate of change of v(t) is less than 1, and therefore that the amplitude of change of v(t) depends only on dt (as one assumes that the variation of epsilon as a wcsvc multivariate normal distribution(N(0,sigma)) is taking into account the stochasticity only). This is strange, as one would expect that this rate of change is controlled by a parameter (i.e. an acceleration) that would control the size of RW steps. Did the authors used dt in place of this acceleration parameter ? Do they consider that this is not necessary as RW are self-similar processes ? and why ? Please explain the logics that drives these choices.

First, for clarity, we reorganized our manuscript to 1): describe our algorithm, 2)emphasize that our goal is to simulate an extreme and simple case of cell expression divergence, with inheritance from mother to daughter cells, and that this algorithm is used at the core of a previously published simulation algorithm for single-cell transcriptomics data (PROSSTT) 3)then we discuss how it can be related to random acceleration process

We could think that since RW are self-similar processes, the overall behaviour would be the same whatever the acceleration parameter, which we then arbitrarily set at 1. However this parameter does matter when put in relation with the number of steps in a cell cycle. Indeed, if these parameters are simultaneously high (a large acceleration parameter with a large number of steps), the divergence would be so fast that the average expression of two daughter cells would be too different from their mother and thus from each other. Our aim was to generate a toy model in which daughter cells inherit a greater part from their mother than their asymmetric part. This set of parameters chosen, acceleration parameter=1,

and nb_steps=3, achieves this. We hope that the revised subsection in Materials and Methods makes the logic clearer **(lines 217-251)**.

p 10 L374-376 and L384-387 say exactly the same thing.

Thank you for pointing this out. We removed this repetition while shortening the ‘Quantification of the mosaic and regulative contributions” section in Results, moving some information into a more extensive introduction in the Material and Methods section.

Fig2: The term "spatial context" is used, while it is not defined in the text. "Spatial" should probably be removed.

We removed “spatial”

p12 L:466 and L467: your 5 configurations contains twice the same entry "unrelated cells": Does this mean that there are actually 4 interesting configurations and not 5 ? Was the text re-read before resubmitting ?

In the former manuscript, one entry is “*unrelated cells* **that are close in the spatial network without being close in lineage nor context**” and the other is “*unrelated cells* **that are close in none of the distance types**”. These are two different entries, which we both called “unrelated cells” for short in the figures, because they were not close in any of the main distances that are related to causal effect we seek to measure (lineage or context).

Since it is misleading we replace by “spatially-close unrelated cells” the cells that are close in the spatial network without being close in lineage nor context **(line 422)**.

p12: "Figure 3D shows that this octant comprises between 10 and 30% of cells along": I understood that each point in these octants is a pair of cells. What means 30% of cells ? Do you mean 30% of cell-pairs ?

Indeed, we meant cell-pairs, this was changed in the revised manuscript, thank you.

p12: could the cells in the Red octant precisely be the L/R matching pairs of cells ? (or contain them). If not, where are the pairs of Left-Right cells ?

To answer the question of whether the red octant (context-only related) is filled essentially with LR matching pairs, we performed the two following analyses.

<u>First analysis - functional symmetry:</u> We extracted the L/R matching pairs in Wormatlas (<u>https://www.wormatlas.org/celllistsulston.htm</u>) by identifying the cells which are named “...L” with a counterpart “...R”, and we kept the pairs that concern our dataset (hermaphrodite,embryonic). This gives 48 pairs of left-right cells. (As a precision, when the paired cells are not present in our dataset but they arise later in development, we test if the respective ancestors up to 3 previous generations are present. If yes, we count the ancestor pairs among the left-right pairs.)

Taking all these 48 identified L/R pairs in wormatlas, we see they occupy a very small portion of the red octant (0.06%). This is partly due to the fact that the functionally identified L/R pairs are late arising cells, whereas the red octant is occupied by pairs of cells from all time points. Nothing allows us to consider the ancestors of symmetric cells A and B as themselves L/R symmetric: these ancestors might very well span very different subtrees, spatial patterns, and leaves fates, with only A and B being functionally identical. We did use this approximation with 3 previous generations but extending it systematically across all time points would be misleading, especially since at early stages the ancestors of A and B may simply coincide.

<u>Second analysis - spatial symmetry:</u> To complement this analysis, we focus only on spatial LR symmetry (without using functionally symmetric pairs). For all the pairs in the red octant (context-only), we compute a spatial left-right asymmetry score and compare it to those of the other octants. The left-right axis is

along the y-axis, with 0 at the center of the embryo. The asymmetry score is S= |y1+y2|+|x1-x2|+|z1-z2|. We see that the pairs in the context-only octant don't display particularly low left-right asymmetry scores.

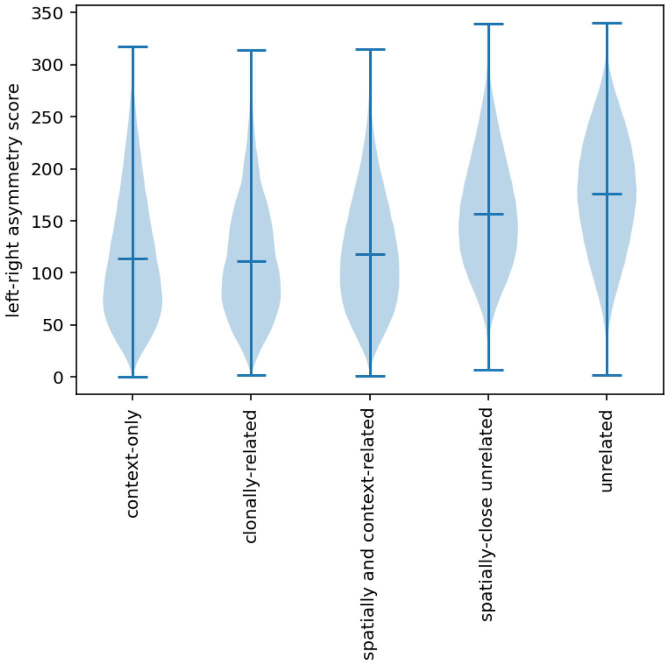

Thus, the cells in the red octant are not composed exclusively of LR matching pairs of cells. We can then answer the second question: in which octants are the LR matching pairs.

We find (histogram below) that they belong in majority to clonally related bin, spatially and clonally related cells, and in 3rd position they belong to context-related bin.

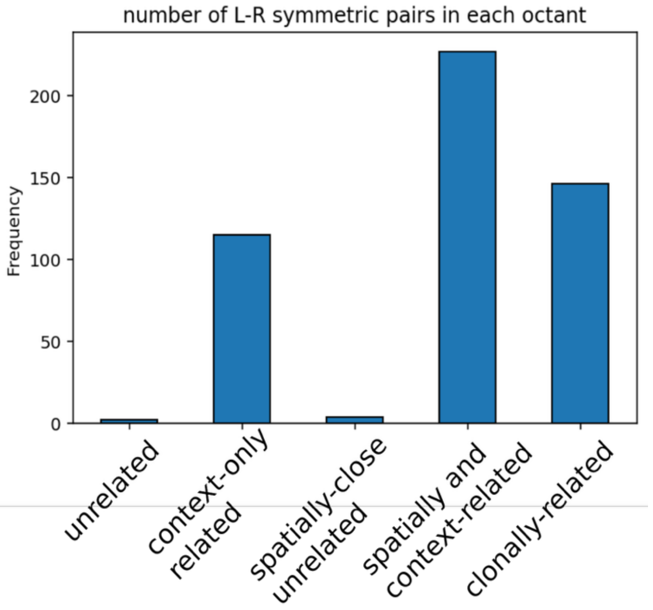

p13: Remark: for pair of cells in the Red octant (similar contexts), does not this suggest that their neighboring cells may have a high probability to be found also in the Red octant (higher than the pairs of cells in other octants).

Yes, indeed, let A and B two cells with similar contexts, and NA and NB given neighbors of A and B, respectively. NA and NB share a subset of neighbors which were part of the contexts of A and B, thus similar, but they also have neighbors (in red and blue, respectively) which might differ. So yes, neighboring cells of a cell pair that have similar contexts are more likely to share similar contexts than a random pair.

A and B share similar contexts

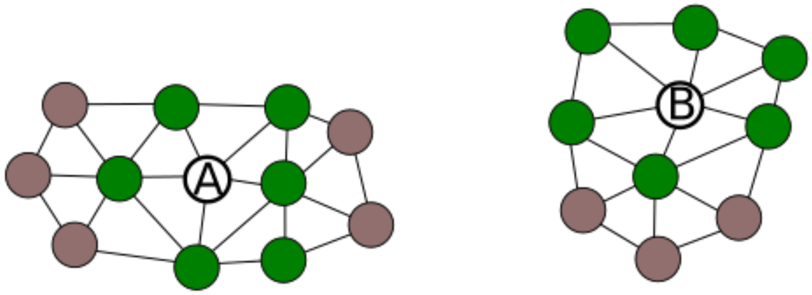

N_A and N_B are neighbors of A and B, respectively.

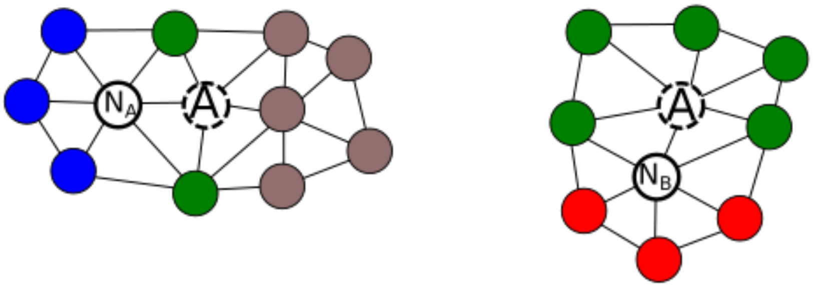

N_A and N_B share a subset of similar neighbors which were already part of A and B's neighbors, but also a subset (in blue and red) which might differ.
